# Right–Left Ventricular Interaction in Left-Sided Heart Failure With and Without Venoarterial Extracorporeal Membrane Oxygenation Support—A Simulation Study

**DOI:** 10.1097/MAT.0000000000001242

**Published:** 2020-08-05

**Authors:** Dirk w. Donker, marko sallisalmi, michael broomé

**Affiliations:** From the * Department of Intensive Care Medicine, University Medical Center Utrecht, Utrecht, the Netherlands; † Anaesthesiology and Intensive Care, Department of Physiology and Pharmacology, Karolinska Institutet, Stockholm, Sweden; ‡ ECMO Department, Karolinska University Hospital, Stockholm, Sweden.

**Keywords:** heart failure, cardiogenic shock, simulation, cardiovascular modelling, right–left heart interaction, mechanical circulatory support, ECMO, venoarterial ECMO

## Abstract

Left ventricular (LV) dilatation is commonly seen with LV failure and is often aggravated during venoarterial extracorporeal membrane oxygenation (VA ECMO). In this context, the intricate interaction between left and right heart function is considered to be of pivotal importance, yet mechanistically not well understood. We hypothesize that a preserved or enhanced right heart contractility causes increased LV loading both with and without VA ECMO. A closed-loop in-silico simulation model containing the cardiac chambers, the pericardium, septal interactions, and the pulmonary and systemic vascular systems with an option to connect a simulated VA ECMO circuit was developed. Right ventricular contractility was modified during simulation of severe LV failure with and without VA ECMO. Left atrial pressures increased from 14.0 to 23.8 mm Hg without VA ECMO and from 18.4 to 27.0 mm Hg under VA ECMO support when right heart contractility was increased between end-systolic elastance 0.1 and 1.0 mm Hg/ml. Left-sided end-diastolic volumes increased from 125 to 169 ml without VA ECMO and from 150 to 180 ml with VA ECMO. Simulations demonstrate that increased diastolic loading of the LV may be driven by increased right ventricular contractility and that left atrial pressures cannot be interpreted as a reflection of the degree of LV dysfunction and overload without considering right ventricular function. Our study illustrates that modelling and computer simulation are important tools to unravel complex cardiovascular mechanisms underlying the right–left heart interdependency both with and without mechanical circulatory support.

## Abstract 2

Supplemental Digital Content is available in the text.

In severe left heart failure, cumulating into refractory cardiogenic shock^1^ and cardiac arrest,^2,3^ venoarterial extracorporeal membrane oxygenation (VA ECMO) is increasingly used as a lifesaving temporary circulatory support modality. It is preferentially applied as a bridge to cardiac recovery but also to chronic mechanical circulatory support or ultimately cardiac transplantation. Systemic perfusion is generally sufficient in VA ECMO, whereas cardiac unloading and more specifically left ventricular (LV) unloading is often unsatisfactory.^4–6^ The most dreadful clinical manifestation of this problem is a massive LV dilatation without aortic valve opening, thrombus formation within the LV cavity, and aortic root accompanied by pulmonary “white-out” due to high left atrial pressures (LAPs) and severe pulmonary edema. Seven percent of VA ECMO cases require immediate LV decompression, whereas a less urgent need for LV unloading is seen in 22% according to Takayama *et al*.^7^ A bundle of adjunct strategies, such as intra-aortic balloon pump,^8,9^ ventriculo-aortic axial pump (Impella),^10,11^ LV venting,^12,13^ and atrial septostomy,^14^ has been shown to be advantageous in handling this significant shortcoming of VA ECMO, and in observational studies, LV unloading has been associated with decreased mortality.^15,16^ Yet, not all patients on VA ECMO are equally affected by LV overloading despite a similar degree of left heart failure, ECMO support flow, and cannulation mode. The pathophysiology and therapies available are thoroughly described by us and others.^5,17,18^ A recent modelling study has shown that this hemodynamic profile of LV overload may be explained by changed loading conditions with a preserved Starling curve in the absence of changes in ventricular contractile function,^19^ while myocardial ischemia, stunning, and the effects of pharmacological therapy should also be considered in clinical reality. From clinical observations, it could be hypothesized that more pronounced LV overload will most likely occur in patients with predominant LV failure and a preserved right ventricular (RV) function as in left proximal acute coronary syndromes. Instead, patients with biventricular failure as encountered in dilated cardiomyopathy, show less pulmonary congestion and LV overload despite a comparable degree of LV failure.^20,21^

Therefore, we hypothesize that this dependency of LV overload on RV contractile function may well equally hold for patients with or without VA ECMO support. Mechanistically, LAP may increase to pathologic levels, when a nonfailing RV pumps blood into the pulmonary circulation, but the failing LV is not capable of handling the RV output due to the high LV afterload and its reduced intrinsic contractile function. Thus, LAP could be considered as a measure of the imbalance in RV–LV interaction, rather than serving as an independent clinical gauge for the degree of LV failure.

Different degrees of combined RV and LV failure is difficult to study both in clinical practice and in experimental models due to a multitude of confounding factors such as varying extension of biventricular disease, autoregulatory responses, and variable loading conditions. Clinical data are difficult to interpret since load-independent measures of LV and RV function are usually not available and in addition patients present with a wide variation in systemic and pulmonary vascular resistances further complicating their interpretation.

Therefore, we developed a simulation model (Aplysia CardioVascular Lab, Aplysia Medical AB, Solna, Sweden, Figure 1), where LV and RV failure can both be simulated including various ECMO support flow rates, while keeping other hemodynamic determinants such as vascular resistance and blood volume constant. Here, we test the hypothesis that preserved RV contractility determines the increase in LAP and LV overload in patients with severe LV failure with or without VA ECMO support in a mathematical simulation model.

**Figure 1.**
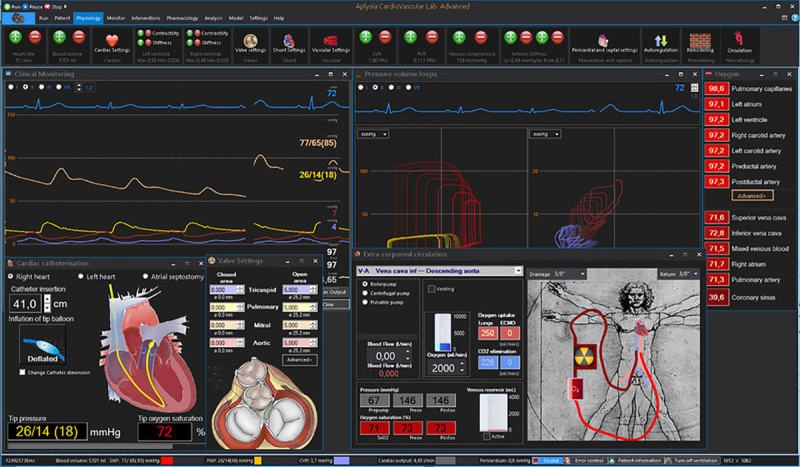
Model interface showing pressure monitoring window, ventricular and atrial pressure–volume (PV) loops, valve areas, pulmonary artery catheter, and extracorporeal circulation windows. Left ventricular PV loops are right-shifted due to systolic heart failure, while left atrial loops indicate an increase in filling pressures.

## Methods

Simulations are performed with a high-fidelity closed-loop real-time model described in detail elsewhere,^5,22–24^ but briefly described below. The model allows gradual changes in LV and RV function and includes optional VA ECMO support. We run simulations with a generic model of LV systolic failure and explore the effects of changing right heart function on clinically relevant parameters representing LV loading conditions.

### Model and Validation

**Figure 2.**
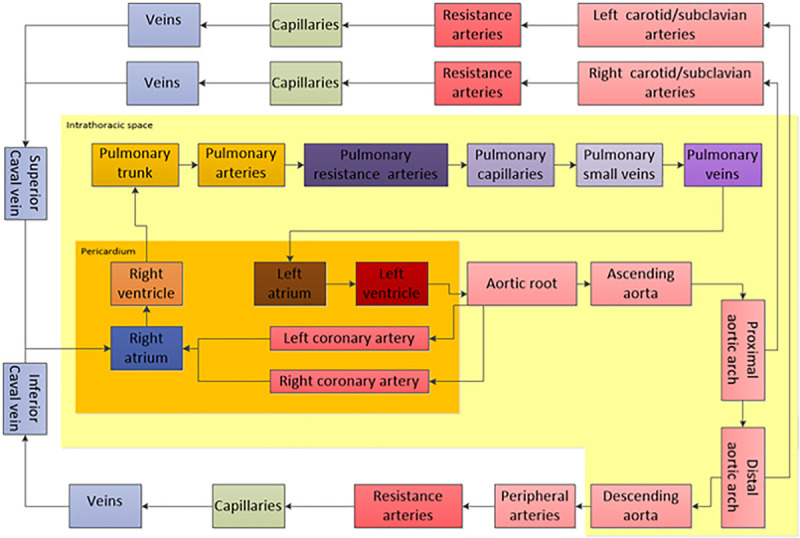
Overview of the hemodynamic model consisting of cardiac chambers inside a pericardium, six pulmonary vascular compartments inside the intrathoracic space and 21 systemic vascular compartments both inside and outside the chest. Model parameters may be modified to simulate heart failure and an ECMO system connected to any vascular compartment.

The Aplysia Cardiovascular Lab model is based on a closed-loop electrical analogue of the cardiovascular system with hydraulic resistance represented by electrical resistance, mass flow inertia by inductance, and the elastic properties of biological tissues represented by capacitances. Known nonlinear relations between pressures, flows, and volumes in the heart and blood vessels are allowed to modify model parameters in every calculation step. All four cardiac chambers are represented by time-varying elastance functions, based on the established fact that the end-systolic pressure/volume ratio of a cardiac chamber is relatively insensitive to loading conditions.^25^ The shape of the elastance function is also relatively insensitive to loading conditions and adopted from other authors.^26,27^ The model also includes a pericardium and 27 vascular segments, including 21 systemic and 6 pulmonary segments (Figure 2). Valves are opening and closing gradually depending on flow and pressure gradients.

Validation of a model describing the entire circulatory system is critically important. Model features and parameters are based on previous publications,^28,29^ physics, and known well-validated physiology models such as time-varying elastance models^26,30,31^ representing cardiac contractility. Details of the model both in normal physiology and heart failure is described in previous publications^5,22–24,32^ and validated based on normal physiology,^33^ well-known heart failure physiology,^34^ and experimental studies.^35^

### Simulation of Isolated Left Ventricular Systolic Heart Failure

**Figure 3.**
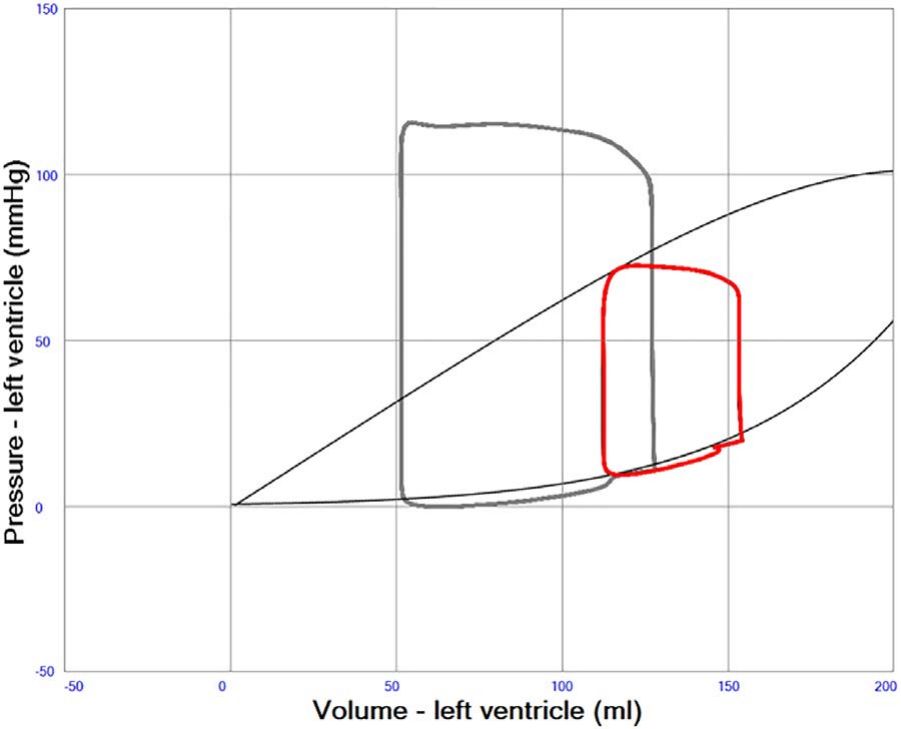
A left ventricular pressure–volume (PV) loop in red illustrating severe systolic left heart failure with an ejection fraction of about 20%. Normal PV loop in gray. End-systolic and end-diastolic pressure volume relations indicated with gray curves. The curved part of the end-systolic line at high volumes represents overstretching of left ventricular myocardium.

The virtual case simulates a patient (70 kg, 170 cm) with a blood volume of 5,561 ml (79 ml/kg) at baseline. LV heart failure was simulated by reducing systolic contractility expressed as the end-systolic elastance from 2.8 to 0.6 mm Hg/ml, resulting in a low cardiac output state with a cardiac index reduced from 2.7 to 1.5 L/min/m^2^. The blood volume was increased by 10 ml/kg to 6261 ml to mimic heart failure pathophysiology including neurohormonal compensatory mechanisms. No autonomic autoregulatory features were included in the simulation, whereas the Starling mechanism is an intrinsic property of the model, therefore an increase in end-diastolic volume results in not only an exponential increase in end-diastolic pressure, but also an increase in end-systolic pressure, partly compensating for the decrease in contractility. See Figure 3.

### Simulation of VA ECMO

The virtual patient was cannulated with venous drainage in the superior caval vein and the arterial cannula inserted into a femoral artery with the tip placed in the descending aorta model compartment. VA ECMO centrifugal pump flow was 3.7 L/min with a rotational speed of 3,600 rpm.

### Modification of Right Heart Function in Systolic Left Heart Failure

RV systolic contractility was varied by stepwise changing the end-systolic elastance between 0.1 and 1.0 mm Hg/ml both up and down from an estimated normal value of 0.7 mm Hg/ml as illustrated in Figure 6 and Table 1.

**Table 1.** Hemodynamic Data in Simulations of Normal Physiology, Left Heart Failure and Left Heart Failure With Variable Right Heart Function. Increases in Right Heart Function Increases Pulmonary Arterial, Pulmonary Capillary, and Left Atrial Pressure as Well as End-Diastolic and End-Systolic Left Ventricular Volumes Both With and Without ECMO

	Heart Rate*/min*	Mean Arterial Pressure, *mm Hg*	LV End-Diastolic Volume, *ml*	LV End-Systolic Volume, *ml*	LV Stroke Volume, *ml*	LV Ejection Fraction, *fraction*	RV Ejection Fraction, *fraction*	Mean Pulmonary Capillary Pressure, *mm Hg*	Mean LA Pressure, *mm Hg*	Mean RA Pressure, *mm Hg*	Mean Pericardial Pressure, *mmHg*	ECMO Flow, *L/min*	Native Cardiac Output, L/min
Normal. No ventilation.	72	102	128	54	77	60%	66%	11.7	6.5	1.7	-0.3	0.00	5.49
Systolic heart failure	72	62	159	125	39	25%	51%	17.7	15.7	3.0	2.3	0.00	2.76
+ 10 ml/kg blood volume	72	68	165	129	41	25%	46%	24.6	22.5	8.6	7.4	0.00	2.87
***No ECMO***													
RV 0.1	72	52	125	102	26	21%	18%	15.6	14.0	11.3	7.0	0.00	1.84
RV 0.2	72	58	140	113	32	23%	24%	18.4	16.0	10.6	7.2	0.00	2.20
RV 0.3	72	62	149	119	35	23%	30%	20.5	18.1	9.8	7.3	0.00	2.45
RV 0.4	72	64	155	123	37	24%	35%	22.0	19.8	9.3	7.3	0.00	2.61
RV 0.5	72	66	159	126	39	24%	39%	23.1	21.0	9.0	7.4	0.00	2.72
RV 0.6	72	67	162	127	40	25%	43%	24.0	21.8	8.8	7.4	0.00	2.81
RV 0.7	72	68	165	129	41	25%	46%	24.6	22.5	8.6	7.4	0.00	2.87
RV 0.8	72	68	166	130	41	25%	49%	25.2	23.0	8.5	7.3	0.00	2.92
RV 0.9	72	69	168	131	42	25%	52%	25.6	23.5	8.4	7.3	0.00	2.96
RV 1.0	72	70	169	132	42	25%	54%	26.0	23.8	8.3	7.3	0.00	2.99
***+ VA ECMO 3600 rpm VCS-DA***													
RV 0.1	72	87	150	144	7	5%	6%	18.4	18.4	8.9	7.4	3.70	0.41
RV 0.2	72	91	161	152	11	7%	12%	21.4	20.9	8.2	7.3	3.69	0.72
RV 0.3	72	93	168	156	14	8%	18%	23.4	22.9	7.7	7.3	3.69	0.92
RV 0.4	72	95	171	159	15	9%	22%	24.8	24.1	7.4	7.3	3.68	1.04
RV 0.5	72	96	174	160	17	10%	27%	25.8	24.9	7.2	7.3	3.68	1.13
RV 0.6	72	97	176	161	18	10%	30%	26.5	25.5	7.1	7.3	3.68	1.20
RV 0.7	72	97	178	162	18	10%	34%	27.0	26.1	7.0	7.3	3.68	1.25
RV 0.8	72	98	179	163	19	11%	37%	27.5	26.4	6.9	7.3	3.68	1.29
RV 0.9	72	98	180	163	19	11%	39%	27.8	26.8	6.8	7.3	3.68	1.33
RV 1.0	72	99	180	164	20	11%	41%	28.1	27.0	6.8	7.2	3.68	1.35

Abbreviations: ECMO, extracorporeal membrane oxygenation; LA, left atrial; LV, left ventricular; RA, right atrial; RV, right ventricular; VA ECMO, Venoarterial ECMO.

### Ventricular and Atrial Septal Properties

**Figure 4.**
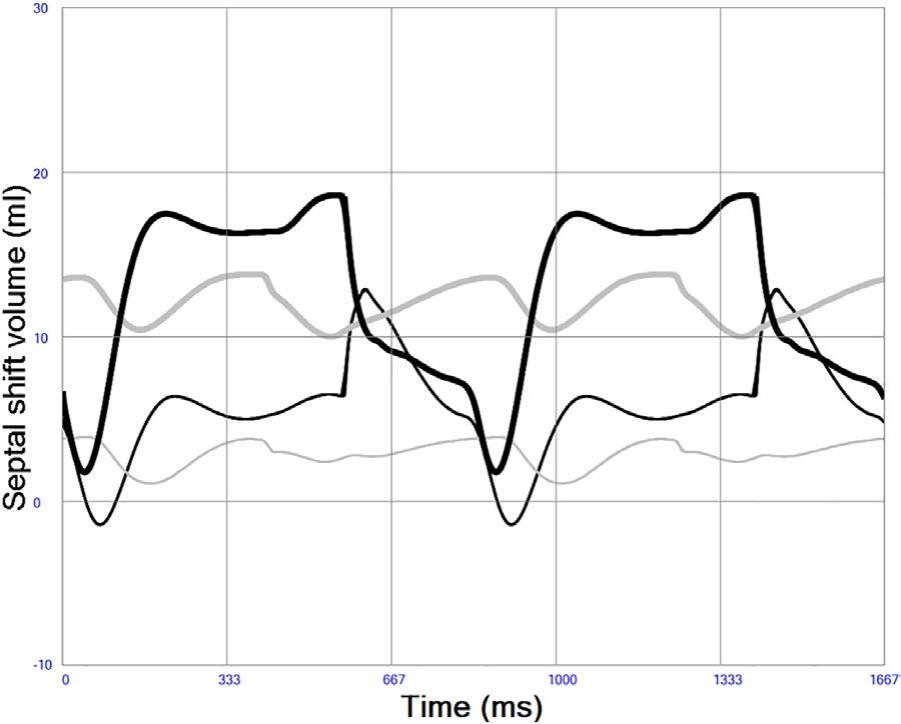
Atrial (thick gray) and ventricular (thick black) septal shift volumes in left heart failure. A positive value denotes shift from left to right, meaning that both the atrial and ventricular septum are shifted slightly to the right during this simulation of systolic left heart failure indicating higher left-sided pressures during the entire cardiac cycle. Less septal shift is seen in normal physiology indicated by atrial (thin gray line) and ventricular (thin black line).

The ventricular septum was simulated as originally proposed by Maughan *et al*.^31^ and adopted by Sun *et al.*^28^ as a septal elastance in addition to the RV and LV elastances in a three compartment model, where both septal shift volume, cross-talk pressures, and gain can be calculated during the entire cardiac cycle. The previous authors used a constant ventricular septal elastance/stiffness (45.9 mm Hg/ml), but the current previously unpublished implementation of the model uses a variable septal elastance where the maximum value is similar to previous authors scaled to the size of the patient. A lower septal stiffness value should be used during diastole, mimicking the fact that the septum is part of the contracting myocardium and therefore more compliant in diastole. Septal elastance/stiffness is therefore set proportional to the mean value of RV and LV elastance/stiffness throughout the cardiac cycle. Atrial septum was similarly simulated with a variable elastance/stiffness depending on the elastance/stiffness of the atria and therefore more compliant than the ventricular septum. See Figure 4.

### Pericardial Properties

**Figure 5.**
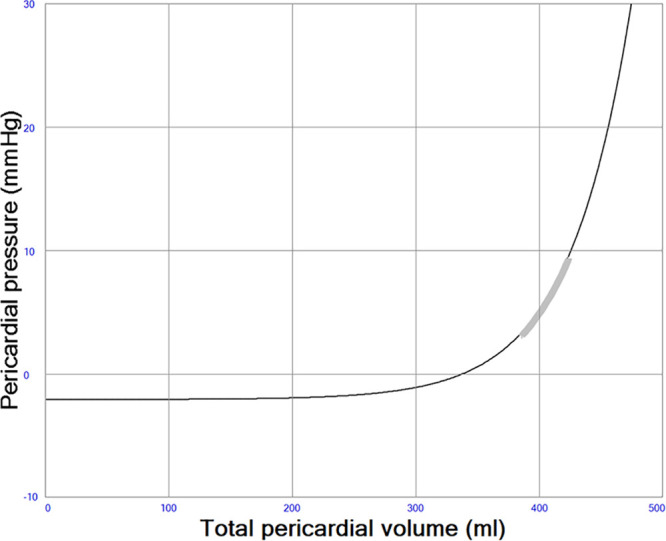
Pericardial pressure–volume (PV) relation. The thick gray line represents the operating range (3–9 mm Hg) in a simulation with acute systolic left heart failure. Pericardial pressures are usually close to zero in normal physiology.

The pericardium was simulated as previously described by Sun *et al.*^28^ as a compliant sac characterized by an exponential pressure–volume (PV) relation containing the four cardiac chambers. The physiologic consequence of this is that the cardiac chambers are competing for space within the sac and that filling acutely may be restricted if chamber dilatation occurs as illustrated in Figure 5. Pericardial pressures are generally positive as usually seen in acute heart failure and shown in Table 1.

### Hemodynamic Variables

Pulmonary arterial and capillary pressures, RAPs and LAPs, arterial blood pressure, and cardiac output were sampled at end diastole. Airway pressures were set to zero to avoid variability due to hemodynamic changes during the respiratory cycle. All hemodynamic variables were sampled at steady-state conditions at least 5 minutes after changing contractility parameters.

### Calculations

The program version used was Aplysia CardioVascular Lab 9.0.1.4 (Aplysia Medical AB, Stockholm, Sweden). Mean values in the model were calculated as weighted running averages with recent values having more impact than older ones. Pressures, flows, volumes, and saturations in every compartment were updated with 4,000 Hz. Hemodynamic differential equations were solved with implicit or explicit Euler’s method. No autoregulatory features or cardiovascular remodeling features were active during simulations.

## Results

### Variable Right Heart Function in Severe Systolic Left Heart Failure Without VA ECMO

**Figure 6.**
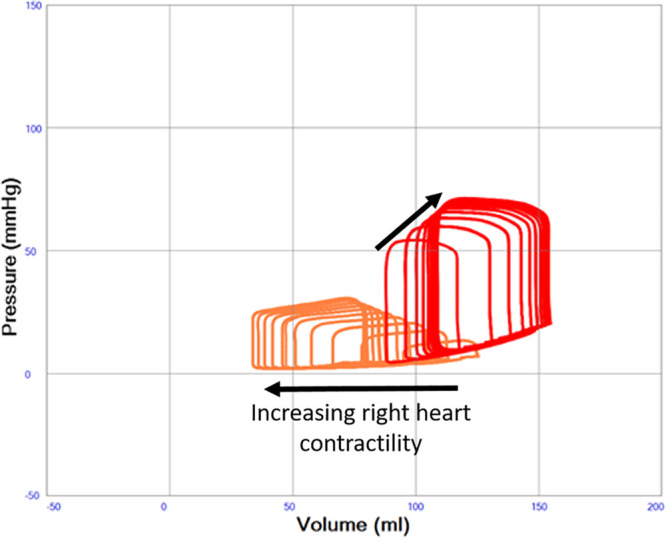
Left ventricular pressure–volume (PV) loops illustrating severe systolic left heart failure (red loops, end-systolic elastance constant 0.6 mm Hg/ml) with variable right heart function (orange loops, end-systolic elastance increasing from 0.1 to 1.0 mm Hg/ml in the direction of the arrow). A progressive dilatation of the left ventricle with increasing end-diastolic pressure is seen with increasing right heart function.

**Figure 7.**
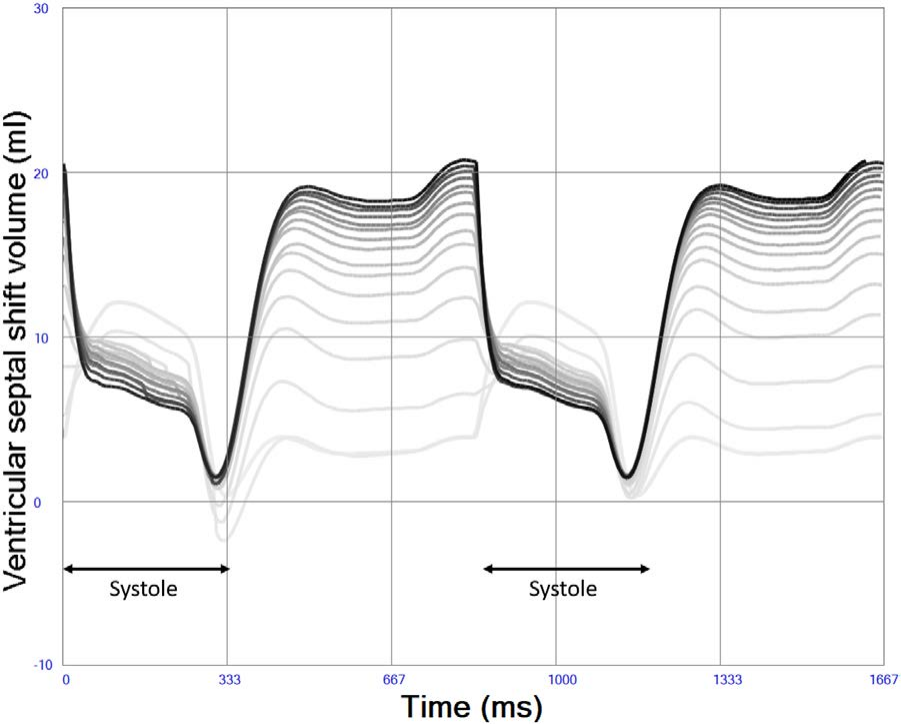
Left-to-right ventricular septal shift volume during increased right heart contractility (indicated by darker lines). Diastolic bulging of ventricular septum from left to right is more pronounced with high right ventricular contractility due to volume loading of the left ventricle, whereas a slight decrease in left-right bulging is seen during systole due to increasing right ventricular pressures.

Native cardiac output increases from 1.84 to 2.99 L/min when right heart contractility increases from end-systolic elastance 0.1 to 1.0 mm Hg/ml in a state of constantly depressed left heart function. Increased right heart contractility also monotonically increases LAP from 14.0 to 23.8 mm Hg, LV end-diastolic (from 125 to 169 ml) and end-systolic (from 102 to 132 ml) volumes as shown in Table 1 and Figure 6. Pericardial pressure remains elevated and increases slightly from 7.0 to 7.3 mm Hg as shown in Table 1. The position of the interventricular septum during diastole bulges progressively more from left to right with increased right heart contractility as shown in Figure 7.

### Variable Right Heart Function in Severe Systolic Left Heart Failure Treated With VA ECMO

**Figure 8.**
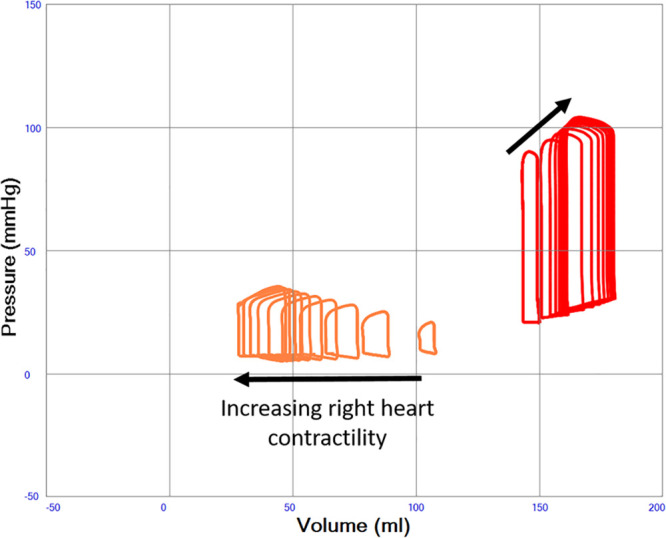
Left ventricular pressure–volume (PV) loops illustrating severe systolic left heart failure (red loops, end-systolic elastance constant 0.6 mm Hg/ml) with variable right heart function (orange loops, end-systolic elastance increasing from 0.1 to 1.0 mm Hg/ml in the direction of the arrow) during VA ECMO support. A progressive dilatation of the left ventricle with increasing end-diastolic pressures is seen with increasing right heart function.

Native cardiac output increases from 0.41 to 1.35 L/min when right heart contractility increases from end-systolic elastance 0.1 to 1.0 mm Hg/ml in a state of constantly depressed left heart function and ongoing VA ECMO. Total systemic blood flow, the sum of native cardiac output and VA ECMO flow, increases from 4.11 to 5.03 L/min. A slight decrease in VA ECMO flow with increased right heart function from 3.70 to 3.68 L/min is seen due to the afterload sensitivity of the simulated centrifugal pump. Increased right heart contractility also monotonically increases LAP from 18.4 to 27.0 mm Hg, LV end-diastolic (from 150 to 180 ml) and end-systolic (from 144 to 164 ml) volumes during VA ECMO as shown in Table 1 and Figure 8. The diastolic position of the interventricular septum bulges progressively more from left to right with increased right heart contractility in a way similar to in Figure 7 without VA ECMO (data not shown). Pericardial pressure remains elevated but decreases slightly from 7.4 to 7.2 mm Hg.

## Discussion

The simulations show that the right–left heart interaction is a major determinant of the loading conditions of the left heart in severe systolic LV failure both with and without VA ECMO. More specifically RV contractility is a major determinant of LAP and should therefore be taken into consideration, when the severity of left heart failure and concomitant LV overload is quantified by measuring filling pressures (See Video 1, Supplemental Digital Content, http://links.lww.com/ASAIO/A531). Our simulations reveal that a preserved or improved right heart function can significantly aggravate pulmonary congestion by increasing left atrial and LV filling pressures. In this sense, right heart contractility will drive LV dilatation and overloading in the setting of VA ECMO.

In previous publications, we have shown that LV unloading during VA ECMO is often unsatisfactory in systolic left heart failure with preserved right heart function.^5,17^ In this study, we have explored the importance of the right heart function in more detail. Although, the clinical importance of the current work needs to be further investigated, our results underscore a pivotal role of RV function in left heart failure. As a consequence, the right ventricle may be considered as a more prominent therapeutic target in left heart failure than anticipated so far, since it acts as a major determinant of LV overload and pulmonary congestion. Likewise, it could be hypothesized that inotropic drug therapy in left heart systolic failure may substantially increase the risk of pulmonary edema and overt decompensation. This may mainly occur when the contractile reserve of the left ventricle is significantly impaired as compared with the right ventricle, as holds for the majority of cases complicated by cardiogenic shock in predominantly left-sided myocardial infarction. Our findings also emphasize the relevance of effective venous drainage during VA ECMO to avoid unwanted right heart output, which in turn increases hydrostatic pulmonary vascular pressure and left heart (over-)loading. Moreover, it has been suggested that the long-term beneficial effects seen with anti-adrenergic, yet negative inotropic, beta-blocking agents in left heart failure,^36^ may in part be due to the negative inotropic effects on the RV myocardium, thereby unloading the left side of the heart. Similarly, the beneficial effects of pulmonary arterial banding in congenital dilated cardiomyopathy^37,38^ may be explained by restricting right heart output, thereby decreasing left heart preload.

From a pathophysiological point of view, this study emphasizes the importance of a serial right–left heart interaction and specifically points to the importance of balancing right and left heart function in heart failure therapy with and without VA ECMO support. This balance is clinically well accessible and can easily be evaluated by visualizing the position of the ventricular septum in a two(four-)-chamber cross-sectional echocardiogram. Here, the dynamics of the septal position should in this context be interpreted as a functional marker of a serial interaction rather than as a causative factor by itself. In this sense, our data indicate that the term “septal function” is a mis-nomer and “septal shift” should rather be considered as a clinical sign of the imbalance between right and LV functional states and their mutual interdependency.

### Limitations

The present 0D model cannot represent three-dimensional (3D) features of the circulatory system. The accuracy of hemodynamic output of simulations in 0D models is however often more reliable than 3D models since it allows more realistic boundary conditions and real-time simulation of the entire circulation including arteries, capillaries, and veins in both the pulmonary and systemic circulation. The cardiac model does not include AV-plane displacement and diastolic elastic recoil, but has despite this proven to produce realistic filling patterns and pressures both in normal physiology and various pathologies.^5,23^

LV and RV filling pressures above 30 and 20 mm Hg respectively may be seen in a clinical VA ECMO population contrasting to lower values in this study. This may be explained by lack of compensatory venous constriction and only a modest fluid infusion in our study aiming to minimize confounding factors. The original model of the ventricular septum used in this study^28,31^ has been further developed by Dickstein *et al.*^39^ who concluded that septal interaction is dependent on right heart volume and curvilinearity of the ventricular PV relation. These effects are not fully taken into account in the current study, but the overall gain of pressure and volume interactions are in the same range as the values found by Dickstein *et al.* and our conclusions therefore remain valid. An experimental isolated heart study by Damiano *et al.*^40^ shows that more than 60% of right heart pressure generation may originate from the left ventricle, but the clinical relevance in an intact heart with a pericardium is unclear. Experimental data are conflicting, but it may be that we have underestimated the right-sided consequences of severe left heart failure to some degree. Furthermore, it should be noted that our approach with a septal stiffness proportional to the mean of left- and right-sided stiffness is a simplification of real life, where septal contractility could be virtually absent or preserved depending on regional myocardial function. A detailed study of septal cross-talk and pathophysiology is beyond the scope of this study.

## Conclusion

The serial right–left heart interaction is of paramount importance in the development of LV dilatation and pulmonary congestion in left heart failure with and without VA ECMO support. It is a preserved or increased RV function that drives the degree of LV (over-)loading. Therefore, LAP cannot be considered as an independent measure of LV dysfunction and myocardial overload without taking the RV function into account. This approach exemplifies that modelling and computer simulation of human hemodynamics are relevant tools to unravel complex cardiovascular mechanisms and identify future therapeutic targets and improve individualized tailoring of VA ECMO, yet further clinical and experimental studies are needed to confirm our findings.

## Acknowledgments

M.B.’s research during the years 2013–2015 was founded by The Swedish Research Council Grant 2012-2800. M.B.’s research during the years 2017–2018 was founded by The Stockholm City Council Grant SLL20160421. In 2019, the research is funded by grant No.20180265 from the Swedish Heart Lung Foundation.

## Supplementary Material


